# Risk factors for pressure sores in adult patients with myelomeningocele – a questionnaire-based study

**DOI:** 10.1186/1743-8454-3-14

**Published:** 2006-12-29

**Authors:** Pål-Erik Plaum, Gunnar Riemer, Kathrine Frey Frøslie

**Affiliations:** 1 TRS – A National Centre for Rare Disorders, Sunnaas Rehabilitation Hospital, N-1450 Nesoddtangen, Norway; 2 Section of Biostatistics, Rikshospitalet-Radiumhospitalet Medical Centre, N-0027 Oslo, Norway

## Abstract

**Background:**

Myelomeningocele (MMC) is a part of a complex neural tube defect and a disorder of the cerebrospinal fluid system. Pressure sores are a frequent complication for patients with MMC. Little is known about the risk factors for pressure sores in adults with MMC. The aim of this study was to investigate an association between the presence of pressure sores and other patient characteristics, in order to develop an improved strategy for the management of sores.

**Methods:**

A structured questionnaire regarding sores, medical condition, function and living factors was designed and sent to the 193 patients with MMC registered in the year 2003 at TRS, a National Centre for Rare Disorders in Norway.

**Results:**

Out of 193 total, 87 patients participated and 71 patients (82%) reported sores; 26 (30%) at the time of the interview and 45 (52%) during the last 5 years. Sores were mostly localized on toes and feet and occurred exclusively in regions with reduced or missing sensibility. A significant association was found between sores and memory deficit (*p *= 0.02), Arnold Chiari malformation (*p *= 0.02) and a record of previous sores (*p *= 0.004). Sores were not significantly associated with hydrocephalus, syringomyelia, nutrition, body mass index, smoking, physical activity, employment or living together with other persons. Some patients (18, 21%) reported skin inspection by others and the remainder relied on self-inspection.

**Conclusion:**

Patients with sensory deficit, memory problems, and Arnold Chiari malformation had a higher risk of having pressure sores. This patient group needs improved skin inspection routines and sore treatment.

## Background

Myelomeningocele (MMC) is a complex congenital spinal anomaly that causes varying degrees of spinal cord malformation, or myelodysplasia. It is a developmental defect in the formation of the neural tube from the embryonic neural plate, and as such is a disorder of the cerebrospinal fluid system. It is commonly referred to as spina bifida, although this term originally described only a malformation of the spine and not of the neurological structures. In this paper we use the term MMC, because it focuses on the neurological structures. In addition, MMC is often associated with tethered cord and an abnormal development of the cranial neural tube, which results in several characteristic CNS anomalies. Hydrocephalus is the most common anomaly, which occurs in more than 90% of infants with MMC [1]. MMC may be associated with the Arnold Chiari malformation, which is characterized by cerebellar hypoplasia and caudal displacement of the lower brainstem into the upper cervical canal through the foramen magnum. This deformity impedes the flow and absorption of cerebrospinal fluid (CSF) and causes hydrocephalus. But not all cases of hydrocephalus are due to Arnold Chiari malformation. Cerebral cortex dysplasia and corpus callosum abnormalities also occur frequently. Patients with MMC present with a spectrum of impairments, but primary functional deficits are lower limb paralysis and sensory loss, bladder and bowel dysfunction, and cognitive dysfunction [1].

Pressure sores are a frequent complication in patients with MMC and they can lead to serious complications such as amputation of the legs and even death. A previous study of the patients registered at TRS, a National Centre for Rare Disorders in Norway, showed that 35% of the patients had pressure sores [2]. Other studies have reported sore incidences per year ranging from 15 to 77% [3-7]. A ten-year prevalence study of adult patients with MMC showed pressure sores in 34% [8]. The causes of pressure sores have been investigated, especially among children. Paraplegia and insensate skin were found to be the main risk factors associated with sores, but mental retardation and hydrocephalus have also been mentioned as risk factors [3,7,9-12]. Two studies related the motor level to sore incidence and localization [7,9], while another study did not find a relation between the spinal lesion level and sores [10]. None of these studies focused exclusively on pressure sores, and only the neurological condition was considered as a causal factor.

For patients with spinal cord injuries, well-established systems exist to evaluate risk factors in contrast to patients with MMC [13]. The aim of this study was to find risk factors for pressure sores in adult patients with MMC. Factors concerning the medical condition and function of the patients, together with skin inspection and living conditions, were investigated.

## Methods

### Study population

The exact number of patients with MMC in Norway is not known. For this study, the patients were recruited from TRS, a National Centre for Rare Disorders in Norway [14]. Registration at the centre is voluntary. Most of the registered patients with MMC had been investigated at the centre during the preceding years and had lumbosacral MMC. By the start of the study in August 2003, a total of 193 patients were registered. All of them were included and received written information about the purpose of the study and the questionnaire. Of these, 87 patients (45%) gave their written consent for participation and completed the questionnaire (Table 1).

**Table 1:** General characteristics of the study population

Parameters	Responders n = 87	Non-responders n = 106	Total TRS population n = 193
Age (mean, SD)	31.8 (9.5)	31.9 (9.9)	31.9 (9.7)
Min/Max (Range)	16/57 (41)	16/67 (51)	16/67 (51)
Female/Male (%)	57 (66%) 30(34%)	55 (52%) 51(48%)	112 (58%) 81(42%)
Height (SD)	1.58 m (12.5)	Not known	Not known
Weight	64.2 kg (13.6)	Not known	Not known
BMI	25.7 kg/m^2 ^(5.1)	Not known	Not known

### Study design

The study followed a cross-sectional design with a questionnaire-based systematic telephone interview. This means, that the patients were interviewed only at one point in time without a follow-up interview. Consequently, risk factors for sores can only be investigated through associations and not as causal factors, which would require a longitudinal effect study.

The questionnaire was designed in collaboration with the Head of the National Spina Bifida and Hydrocephalus Users' Organization, both concerning the topics and in the formulation of questions [15]. The questionnaire was not formally validated. It contained a total of 225 questions on five pages. The questions required mostly "yes/no" answers, although some of them contained several categories. Most of the topics were assumed to be potential risk factors for pressure sores.

The essential topics of the questionnaire are summarized in Table 2. The last part of the questionnaire allowed for open comments about personal experience of sores and individual recommendations concerning their prevention. The questionnaires were sent to the participants two weeks in advance of the telephone interview. The participants were encouraged to collect information concerning the questions from their families and doctors. Both the patient and the interviewer had the questionnaire at hand during the interview. Most of the patients had been at the centre several times and were known by the interviewer, Pål-Erik Plaum. The interviews were performed between August 2003 and December 2004.

**Table 2:** The table contains the specific parameters to which the 87 patients responded.

**Parameters**	**Yes**	**No**	***p*-value**
**Living factors**			
Living together with other persons	44 (50%)	43 (50%)	n.s.
In employment	19 (22%)	68 (78%)	n.s.
Driving car	57 (66%)	30 (34%)	n.s.
Consciousness of nutrition	40 (46%)	47 (54%)	n.s.
Smoking	19 (22%)	68 (78%)	n.s.
Regular physical activity	36 (41%)	51 (59%)	n.s.
Physiotherapy more than 12 times per year	32 (37%)	55 (63%)	n.s.
**Mobility function**			
Mobile without aids	28 (32%)	59 (68%)	n.s.
Standing without aids	37 (43%)	50 (57%)	n.s.
Mobile with crutches	27 (31%)	60 (69%)	n.s.
Using orthosis	16 (18%)	71 (82%)	#
Permanent wheelchair user	49 (56%)	38 (44%)	n.s.
**Sensory function**			
Reduced or missing sensibility	83 (95%)	4 (5%)	#
**Stoma/Diapers**			
Appendicostomy	16 (18%)	71 (82%)	n.s.
Urostomy	30 (34%)	57 (66%)	n.s.
Colostomy	12 (14%)	75 (86%)	#
Diapers	49 (56%)	38 (44%)	n.s.
**Neuropsychological deficits**			
Memory deficit	44 (51%)	43 (49%)	0.02
Learning difficulties	52 (60%)	35 (40%)	n.s.
Concentration difficulties	54 (62%)	33 (38%)	n.s.
Speech deficit	5 (6%)	82 (94%)	#
Simultaneous capacity deficit	55 (63%)	32 (37%)	n.s.
Orientation problems	44 (51%)	43 (49%)	n.s.
**Additional neurological diagnosis**			
Hydrocephalus	58 (67%)	29 (33%)	n.s.
Shunt	54 (62%)	33 (38%)	n.s.
Syringomyelia	9 (10%)	17 (20%)	#
Tethered cord	45 (52%)	17 (20%)	n.s.
Surgical treated Arnold Chiari malformation	16 (18%)	71 (82%)	0.02
**Sore management**			
Previous sores/current sores	45/26 (52/30%)	16 (18%)	0.004
Skin inspection by others	18 (21%)	69 (79%)	n.s.
Plastic surgery	15 (17%)	72 (83%)	n.s.

When the sum of the "Yes-" and "No-column" was less than 87 (100%), the parameter was unknown for the participant. The last column presents p-values for associations between the parameters and sores. Remarks: n.s. = not significant; # = p-values were not calculated due to low numbers.

### Definition of pressure sores

The "European Pressure Ulcer Advisory Panel" defines pressure sores as an area of localized damage to the skin and underlying tissue caused by pressure, shear, friction and/or a combination of these [16]. Pressure sores are classified as follows: grade 1: non-blanchable erythema; grade 2: abrasion or blister; grade 3: superficial ulcer; grade 4: deep ulcer. The questionnaire inquired solely about skin alterations corresponding to grades 3 and 4, because the mild skin alterations were assumed to be difficult for the patients to recognize.

### Definition of the neurological impairment

The medical condition and function of the participants were assessed in terms of mobility and sensory function, the presence of stoma, the use of diapers, neuropsychological function and the presence of additional neurological diagnoses. The most apparent neurological impairment of MMC is lower limb paralysis and sensory loss. However, it was not possible to examine the patients neurologically and consequently the exact anatomical level of the lesion could not be obtained. To acquire general information about the mobility a number of questions were asked (Table 2). Concerning the sensory function the patients were asked if they had reduced or missing sensibility from buttocks to knee, from knee to toes, or on the whole leg, both right and left. Because the additional neurological diagnoses (tethered cord and Arnold Chiari malformation) are often asymptomatic and not known to the patients, we asked whether they had surgical treatment for these conditions.

### Statistical methods

The patients were categorised in three groups: group (1) reporting sores at the time of the interview, group (2) reporting sores in the last five years, but not at the time of the interview, and group (3) reporting of never having had sores. The localization and the duration of the sores in group (1) were recorded.

Associations between potential risk factors and sores, as categorised in these three groups, were tested using contingency tables and the Pearson chi-squared test. A *p*-value of 0.05 or less was considered statistically significant. No correction for multiple testing was done.

### Ethics

The study was approved by the Regional Committee for Medical Science Ethics and the National Organization of Spina Bifida and Hydrocephalus [17].

## Results

### Description of the study population

**Figure 1:**
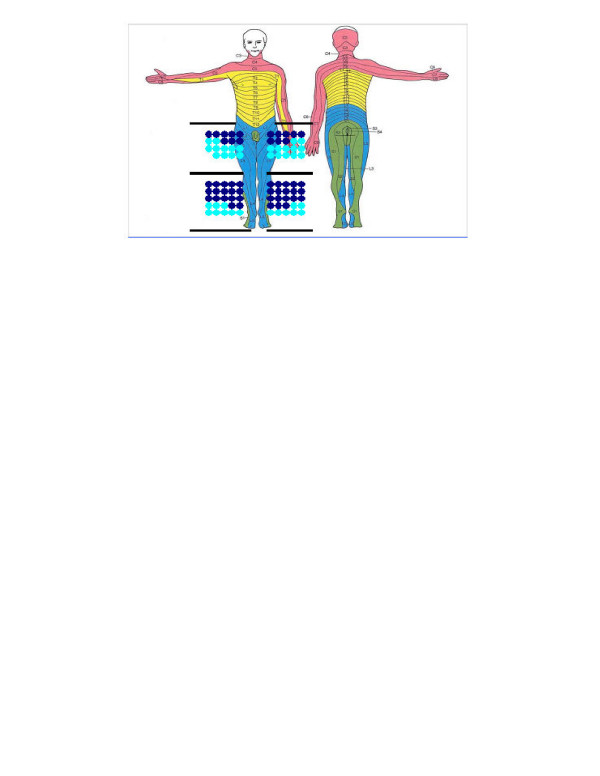
Localization of sensory deficits (n = 87 patients). The lower limbs were divided into four regions: left and right leg with an upper region reaching from buttocks to knee, and a lower region from knee to toes. Light blue spots indicate reduced sensibility and dark blue spots missing sensibility. One patient could have several regions with reduced or missing sensibility. The spots were only marked in relation to the body showing the ventral perspective.

The general characteristics of the 87 participants, of the non-responders and of the total population are presented in Table 1. No significant differences in age and gender distribution were found between the participants and the non-responders. Information concerning the living factors, medical condition, function, and some aspects of sore management is summarized in Table 2. The main findings are that 50% of the patients lived alone, 78% were unemployed, and that 56% were permanent wheelchair users. Sensory deficits, reported in 95% of the study group, were most obvious distally in the legs and symmetrically distributed (Figure 1). Concerning the neuropsychological deficits, 63% of the patients had simultaneous capacity deficit, 62% had concentration difficulties, 60% had learning difficulties, 51% had memory deficit, 51% had orientation problems and 6% had speech deficit. Shunted hydrocephalus was present in 62% of the patients, and 18% had surgically treated Arnold Chiari malformation.

Sores were present at the time of the interview in 26 patients (30%; 95% CI = 20 – 40%), present in the last 5 years, but not at the time of the interview in 45 patients (52%; 95% CI = 41 – 62%), and 16 patients reported never having had sores (18%; 95% CI = 11 – 27%).

### Associations between potential risk factors and sores

In all cases, sores were associated with the degree of sensory deficit: 19 patients reported sores in regions with missing sensibility, 7 patients reported sores in regions with reduced sensibility, and none of the patients reported sores in regions with normal sensibility. The significant associations are shown in Table 2. Pressure sores were significantly associated with memory deficit and with surgically treated Arnold Chiari malformation, *p *= 0.02 for both associations. Another significant association was found between previous sores and current sores, *p *= 0.004: all the 26 patients with current sores had also had sores at some point in the previous five years. The other parameters gave no significant association.

### Skin inspection

All the 87 patients reported that they inspected their skin themselves to see if they had sores. However, 18 patients (21%) also had other people who inspected their skin, 11 were family members and nine were health professionals (two of the patients had skin inspection by both, family members and professionals).

### Sore characteristics and treatment

**Figure 2:**
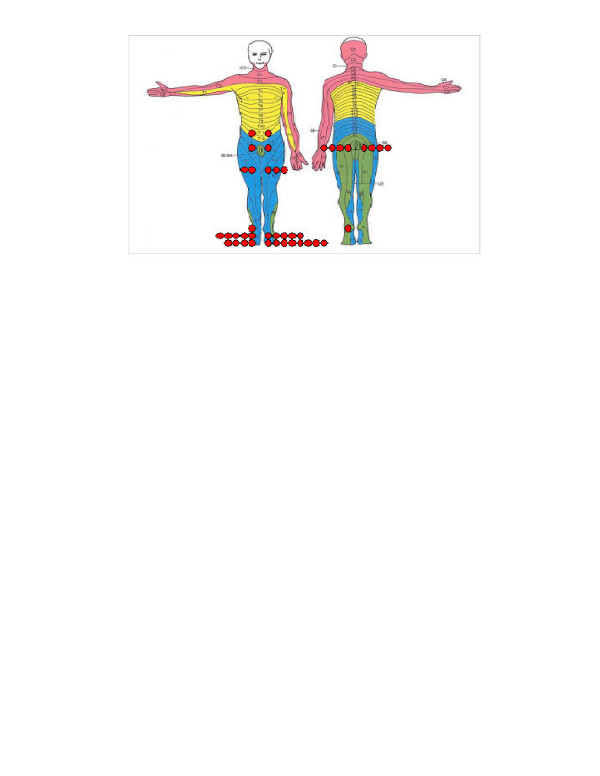
Localization of pressure sores (n = 26 patients). Every spot indicates the location of one sore. When there was more than one sore in a region, the spots were aligned to the side of the body image.

**Figure 3:**
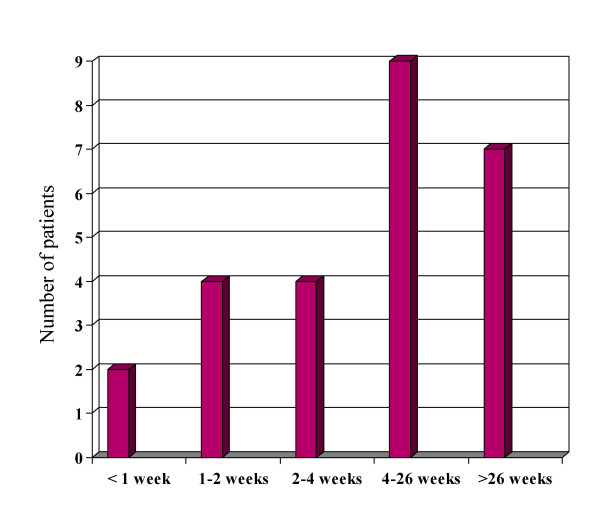
Duration of pressure sores (n = 26 patients).

Out of the 26 patients with a total of 41 current sores, 16 had one sore, seven had two sores, one had three sores, and two had four sores (Figure 2). There were 12 sores located on toes, 10 on feet and eight on buttocks. Other localizations were rare. The duration of the sores varied from less than one week to longer than 26 weeks and most of the sores lasted longer than one month with many longer than six months (Figure 3).

The sores were treated in 13 cases by the patients themselves, in 12 cases by professionals and in 5 cases by family members. Consequently, a few cases were treated by several persons. Sore treatment was done daily for 20 patients, three were treated every second day and three had weekly treatment. Out of the 71 patients having sores both currently and previously, 23 had outpatient follow-up because of previous sores and seven due to current sores, 26 (37%) had been examined by a plastic surgeon and 13 (17%) patients with previous sores and two with current sores had undergone surgery. Many of the patients had received follow-up over many years. Patients were encouraged to note their personal experiences for treatment and avoidance (Table 3). It was noticed that the sores in some patients were localized several times at the same place. Several patients reported less sores while using penicillin for treatment of a urinary tract infection.

**Table 3:** Typical personal comments from the patients concerning their sores

**Comments on sore development and localization:**
"I had a sore five times at the same spot on my buttocks in a two year period. The sore resulted in osteomyelitis needing operation."
"I had 7 operations of one sore during a two year period."
"I had sores under my toes which spread gradually up to my knees during a period of 14 years. Finally both legs had to be amputated."
"I developed a sore after a bulla on the sole."
"I developed a sore under a urostomy plaster."
"I developed a sore on my back after a tethered cord operation."
"I developed a sore under the plaster after a femur fracture."

**Recommendations concerning sore treatment and prevention:**

"Use wool stockings and avoid seams and friction of clothes."
"Inspect feet by means of a mirror."
"Contact experienced podiatrist regularly."
"Use adapted shoes."
"Use calendula cream against hard and ruptured skin."
"Apply Aloe vera or honey into the sore."
"Change sitting position regularly."

## Discussion

### Study population

For age and gender, no significant differences were found between the participants and the non-responders. In the registry used for this study, there was no systematic data on neurological function, the level of the MMC or the presence of additional neurological diagnoses. However, in a previous study, the prevalence of the additional neurological diagnoses of MMC in Norway was 64% for shunted hydrocephalus, 13% for syringomyelia, 80% for tethered cord and 43% for Arnold Chiari malformation [18]. This indicates that the study population used here was representative for the patients with MMC in Norway concerning the severity of the condition. From the reported distribution of sensory and motor deficits, it seems likely that all the patients had lower thoracic or lumbosacral myelomeningocele.

### Sore prevalence

The prevalence of sores at the time of the interview found in this study corresponds with previous findings in Norway from 1996 [2], where the sore prevalence was estimated to be 35% and to a Spanish multicenter study from 1993, where the sore prevalence was 31.7% [5]. Variations in sore prevalence in different studies may be explained by differences in population age, different inclusion criteria, and different target groups by the medical care systems [3-8].

### Reliability of the data

All the data were acquired through a questionnaire-based interview. To help with reliability, the questionnaire was sent prior to the interview and the patients were encouraged to gather information before the interview was performed. A weakness of the study is that self-reporting was used to describe clinical parameters, and that the data were not validated. Therefore statements on associations between clinical factors and sores should be interpreted cautiously. The *p*-values of the associations found between sores and memory deficit and surgically treated Arnold Chiari malformation were not very strong and they have not been adjusted for multiple tests. If a physical examination had been used, the data would have been more reliable.

### Sore risk factors

The aim of this study was to identify risk factors for sores. Sores were invariably associated with sensory deficit. This finding is not unexpected because reduced superficial sensation increases the risk of mechanical and thermal skin damage. Impairment of the autonomic innervation alters blood circulation, temperature homeostasis and healing processes. This could explain why sores were mostly localized on feet and toes. This corresponds with a previous study, which showed sores on the plantar surface of the metatarsal heads and of the heel in 75 and 64%, respectively, and sores were related to the absence of plantar sensation [11].

Mobility function was not associated with sores in contrast to other studies, where the motor level was related to sore incidence [7,9]. This may be because the questions on mobility function were insufficiently precise.

Among the cognition deficits recorded, sores were associated with memory deficits. This seems evident because intact memory function is essential for skin inspection routines, sore treatment, and prophylactic actions such as regular "sit-ups". Only five patients reported problems with speech. From the literature, it is well known that individuals, mostly children, with MMC have a certain cognitive profile, characterised by reduced memory, cognitive processing, arithmetic, visual perception/construction and fine motor coordination [19-24], but also with approximately normal verbal ability [19,20,23,25].

Of the additional neurological diagnoses often occuring with MMC, sores were significantly associated only with surgically treated Arnold Chiari malformation. On the other hand, somewhat unexpectedly, hydrocephalus, either shunted or not, gave no association. This can be explained by recent research showing that patients with MMC and Arnold Chiari malformation seem to have a distinct cognitive profile characterised by impaired visual analysis and synthesis, verbal memory, and verbal fluency [26,27].

Surprisingly, our study found no associations between sores and living factors such as consciousness of nutrition, smoking, living together with other persons, employment, regular physical activity and physiotherapy. Nor were gender, age and body mass index associated with sores.

### Sore management

All the patients with current sores had also had sores at some point in the last five years. This indicates that patients with current sores often have a history with repeated sores. The high prevalence rate over several years could indicate insufficient diagnosis and treatment. In the literature, recurrence rates greater than 80% are reported which led to a new approach with an education program in personal skin and self-care after reconstructive sore surgery that could reduce sore recurrent rate to 25% [28]. In this context, the comments about personal experiences revealed that sores typically were on the same area and emerged gradually with the risk of becoming chronic and more severe. The patients' recommendations for the sore management show that many of them have been informed about useful strategies. To what degree these activities were performed and were effective is unknown. Possibly improved skin inspection, especially by other persons, might contribute to a reduced prevalence.

## Conclusion

The MMC patients with sensory deficit, memory deficit, surgically treated Arnold Chiari malformation and previous sores had a significantly higher risk of having current pressure sores. The association of sore prevalence with sensory deficit as a risk factor for sores is in accordance with other studies. New findings for this study are that sores were associated with memory deficit and surgically treated Arnold Chiari malformation. Because pressure sores are a life-threatening condition, there is a need for improved skin inspection routines and treatment in patients with this profile. Further research should focus on these risk factors in order to improve sore prevention.

## Competing interests

The author(s) declare that they have no competing interests.

## Authors' contributions

All authors have read and approved the final version of the manuscript.

PP: elaboration of the questionnaire, performing the interviews, acquisition and revising the data, writing the article

GR: elaboration of the questionnaire, gathering and revising the data, writing the article

KFF: performing the statistical analysis, participation in writing the article
